# Immunological consequences of kidney cell death

**DOI:** 10.1038/s41419-017-0057-9

**Published:** 2018-01-25

**Authors:** Maysa Sarhan, Anne von Mässenhausen, Christian Hugo, Rainer Oberbauer, Andreas Linkermann

**Affiliations:** 1 Division of Nephrology and Dialysis, Department of Medicine III, Medical University Vienna, Vienna, Austria; 2 Division of Nephrology, Department of Internal Medicine III, University Hospital Carl Gustav Carus at the Technische Universität Dresden, Dresden, Germany

## Abstract

Death of renal cells is central to the pathophysiology of acute tubular necrosis, autoimmunity, necrotizing glomerulonephritis, cystic kidney disease, urosepsis, delayed graft function and transplant rejection. By means of regulated necrosis, immunogenic damage-associated molecular patterns (DAMPs) and highly reactive organelles such as lysosomes, peroxisomes and mitochondria are released from the dying cells, thereby causing an overwhelming immunologic response. The rupture of the plasma membrane exhibits the “point of no return” for the immunogenicity of regulated cell death, explaining why apoptosis, a highly organized cell death subroutine with long-lasting plasma membrane integrity, elicits hardly any immune response. Ferroptosis, an iron-dependent necrotic type cell death, results in the release of DAMPs and large amounts of lipid peroxides. In contrast, anti-inflammatory cytokines are actively released from cells that die by necroptosis, limiting the DAMP-induced immune response to a surrounding microenvironment, whereas at the same time, inflammasome-associated caspases drive maturation of intracellularly expressed interleukin-1β (IL-1β). In a distinct setting, additionally interleukin-18 (IL-18) is expressed during pyroptosis, initiated by gasdermin-mediated plasma membrane rupture. As all of these pathways are druggable, we provide an overview of regulated necrosis in kidney diseases with a focus on immunogenicity and potential therapeutic interventions.

## Key Points

- Regulated necrosis is a genetically determined process that contributes to acute kidney injury and causes antibody-mediated rejection (ABMR).
- Necroptosis, ferroptosis and pyroptosis are the best-studied pathways of regulated necrosis in acute kidney injury and transplantation.
- Necrotic cell death results in the release of DAMPs and is often prone to elicit an immune response.
- Failure to efficiently remove necrotic debris by LC3-associated phagocytosis (LAP) results in autoimmunity.
- During the process of kidney transplantation, necrotic cells are capable of priming memory B cells that may drive ABMR years after transplantation.
- Necroptosis, ferroptosis and pyroptosis can be therapeutically interfered with by small molecules.

## Introduction

Regulated cell death (RCD) is defined by a genetically encoded program that results in cell death by either apoptosis or necrosis. Cell death is referred to as “programmed” if it occurs during physiological development^1^. Whereas the plasma membrane integrity is maintained during “apoptosis”, it ruptures in “necrosis”^1^. Several mechanistic insights into RCD have been achieved in the last 5 years^2–13^, following the detection of receptor-interacting protein kinase 3 (RIPK3) as a mediator of necroptosis, the prototype pathway of regulated necrosis (RN). Before this important discovery, cell death has been used almost synonymously with apoptosis. The most critical difference between apoptosis and RN most likely is the release of damage-associated molecular patterns (DAMPs) that drive immunogenicity^14^, a process that is absent in apoptosis. It emerged that autoimmunity is caused by DAMP release following RN, and the question has finally been asked if there is any inflammation that is not a consequence of necrosis to some extent – answers to this question are hard to provide, and it may cause a long standing debate^15^. Intriguingly, the pathways of RN can be therapeutically targeted^2,6,16–18^, resulting for the first time in one and a half centuries of medicine in the awareness of necrosis as a pathophysiological principle and the chance to finally treat such disorders. Given that RCD has been reported in several eukaryotic organisms, and ferroptosis has recently been suggested to happen in *Arabidopsis*^19,20^, obviously, we are discussing pathways of tremendous evolutionary conservation, and thus of outstanding importance.

Among the pathways of RCD, some are critical for the development of an organism and are therefore referred to as “programmed” cell death (PCD). The only known form of PCD is apoptosis, but it was suggested that during physiological processes, necrosis might represent the mechanism of turnover, for example, during involution of the mamma following lactation and the obliteration of the Mullarian duct^21^ – if the detection of these mechanisms would succeed, these processes should be referred to as “programmed necrosis”. Moreover, one example for programmed necrosis may indeed exist. In *c. elegans*, there is an example of the spike cell that undergoes nonapoptotic cell death as a means of normal development^22^.

In contrast, RN defines all genetically encoded pathways of cell death that result in plasma membrane rupture, regardless if the trigger comes from the inside of the cell (e.g., in ferroptosis), the outside of the cell (e.g., in necroptosis and pyroptosis) or if it is the result of a distant means of cell death induction that results in plasma membrane rupture (as in complement-mediated lysis). The terms “ferroptosis”, “necroptosis” and “pyroptosis” refer to defined RN subroutines, which will be introduced here, alongside with apoptosis. It is understood that more signalling pathways of RN may exist, such as parthanatos and mitochondrial permeability transition-mediated RN (MPT-RN). However, for this review, we will subjectively focus on apoptosis, necroptosis, pyroptosis and ferroptosis. Today, the first clinical trials in phase 2 are recruiting patients to analyze the therapeutical potential of cell death inhibitors as newly generated first-in-class compounds, such as GSK2982772, a necrostatin (Nec) and receptor-interacting protein-1 (RIP1) kinase inhibitor (clinical-trials.gov identifier: NCT02903966).

## A conservative introduction to RCD

### Apoptosis – well defined and well tolerated by the immune system

During normal development, apoptosis is by far the dominating pathway of RCD, meeting the definition criteria for PCD as it is important for development and inevitably has to happen in any given organism^1,23^. The details of the signaling pathway of apoptosis have been extensively reviewed elsewhere^24^. Very briefly, two major subroutines, the extrinsic death receptor (DR)-mediated pathway and the intrinsic pathway that involves mitochondria^25^, result in the typical morphological changes that include shrinkage of the cells following subsequent plasma membrane blebbing and exposure of phosphatidylserine (PS) to the outer leaflet of the plasma membrane. Macrophages, NK cells and others sense the PS exposure on the surface as an eat-me signal. This complex intercellular sensing has not been entirely understood, but recent discoveries have identified several important components such as a PS receptors^26^, Ucp2^27^ and intracellular messengers like ELMO1 with its downstream partner Rac1^28^. Consequently, apoptotic cells are rapidly cleared within the healthy organism in a process referred to as efferocytosis, the process by which dead or dying cells are engulfed and digested by phagocytes^29,30^.

### Ferroptosis – ancient necrosis by lipid peroxidation

**Fig. 1:**
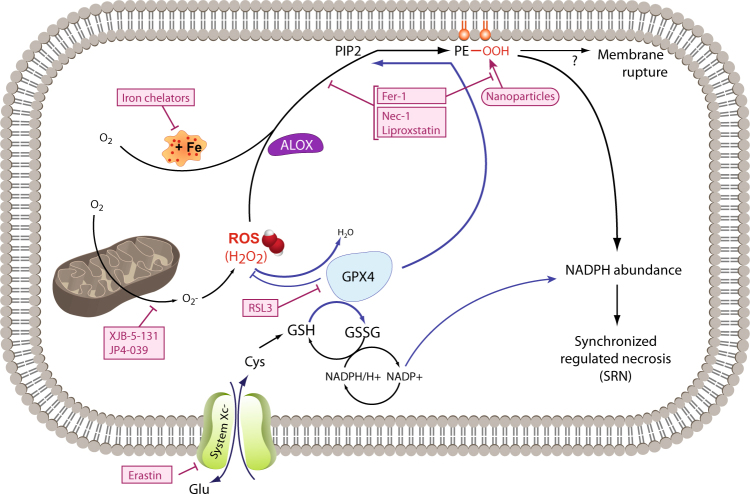
The signalling pathway of ferroptosis Peroxidation of membrane lipids, predominantly phosphatidylinositole and phosphotidylethanolamine, represents the point of no return during ferroptosis that results in loss of NADPH abundance and synchronized regulated necrosis (SRN) in sensitive organs, such as the renal tubular compartment or the myocardium. Lipoxygenases (ALOX) mediate lipid peroxidation, predominantly and specifically of PIP2 and phosphatidylethanolamine (PE). The constitutively active function of glutathione peroxidase 4 (GPX4), a selenoenzyme that requires glutathione (GSH) to function, prevents lipid peroxidation. Ferroptosis may be triggered by inhibition of system Xc-minus, a cys/glu-antiporter in the plasma membrane by a lethal compound referred to as erastin. Inhibition of system Xc-minus functionally inhibits the activation of the GSH-synthase (GSSG), resulting in GSH depletion, dysfunction of GPX4 and ferroptosis. RSL3 induces ferroptosis directly by inhibition of GPX4, and certain nanoparticles are capable of inducing ALOX activation *in vitro*. Before lipid peroxidation occurs, it may be prevented by the ferrostatins liproxstatin, ferrostatin-1 (Fer-1), necrostatin-1 (Nec-1), and the novel compounds 16–86, XJB-5-131 and JP4-039. The latter carries intrinsic anti-necroptotic activity beyond its function as a ferrostatin. Ferroptosis accounts for the majority of tubular cell loss during acute kidney injury because of its mechanism of SRN that shuts down an entire functional unit, but ferroptosis may be triggered by any cell death that occurs in cells of the functional syncytium because of the preterminal loss of NADPH that occurs in all cell death pathways

As demonstrated in Fig. 1, “free” or “labile” iron has a central role in ferroptosis, hence the name. Iron loses its oxidative capacity when bound to ferritin, and iron chelators were among the first ever compounds that reversed renal tubular injury induced by diverse means^31–34^, and labile iron is a known risk factor to develop acute kidney injury (AKI) in clinically relevant settings^35,36^. Desferoxamin has become a standard control agent for AKI when induced *ex vivo* in settings such as isolated renal tubules or *in vivo* in models of acute renal failure^37^. In those days, the term ferroptosis has not been used, as it was introduced by Dixon et al. in 2012^2^, but it is rather obvious that some of these articles have described overlapping phenomena. Today, the definition of ferroptosis may best be characterized by “a subroutine of regulated necrosis that depends on lipid peroxidation, mediated predominantly polyunsaturated fatty acids (PUFAs)^38^.”

AKI and renal cancer are very commonly affected by ferroptosis. As it appears, there is a determination of ferroptosis to this particular tubular tissue that results in synchronized RN (SRN) when the redox potential, referred to as the nicotinamide adenine dinucleotide phosphate (NADPH) abundance^39,40^, is lost. Importantly, ferroptosis in AKI induced by cisplatin toxicity has been well established^41^ in addition to the well-described role in ischemia–reperfusion injury (IRI)^42,43^.

The key players in the ferroptosis pathway are glutathione peroxidase 4 (GPX4) and arachidonate lipoxygenases (ALOX), the latter of which are believed to mediate lipid peroxidation (see Fig. 1). In fact, the labile iron pool is controlled by a third important enzyme, the phophorylase kinase G2, which modulates the sensitivity to ferroptosis^38^. PUFA and phosphatidylinositol-4,5-bisphosphate (PIP2) peroxidation results in lipid peroxidation and loss of NADPH abundance downstream of this event. GPX4, a selenoprotein, which employs glutathione (G-SH) to reduce H_2_O_2_ to GS-SG and H_2_O, represents the essential opponent of ALOX activation and requires glutathione to function. In addition, GPX4 explains why selenoproteins are required for mammalian survival in genereal^44^. GSH rate-limiting components are provided by a glu/cys antiporter in the plasma membrane, which is referred to as system Xc-, and the blockade of which by a small molecule termed erastin inevitably depletes GSH levels, results in GPX4 dysfunction and ALOX-mediated lipid peroxidation and ferroptosis. Similarly, GPX4 can directly be targeted by another lethal compound referred to as RSL3. Obviously, genetic loss of GPX4 also results in failure to prevent spontaneously occurring ferroptosis and embryonic lethality in mice^45–47^.

System Xc- consists of the two subunits, SLC3A2 and SLC7A11, the latter being expressed under the control of p53^48^ (a tumor-suppressor gene, flanked by ALOX genes). Given the well-described beneficial effects of p53-deficient mice in several models of AKI and even renal transplantation employing small interfering RNA (siRNA) that targets p53, the protection might result either from resistance to ferroptosis (e.g., in the siRNA experiments) or from an overlapping conventional knockout that might affect flanking genes in the genetic models^49–51^.

### Necroptosis – MLKL-mediated necrosis to defend viruses

**Fig. 2:**
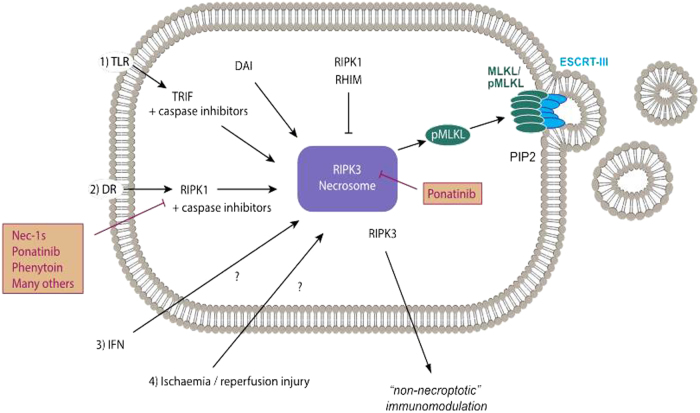
Necroptosis Phosphorylated mixed linage kinase domain like (pMLKL) is the only known mediator of necroptosis and its detection defines the activation of this pathway. Potentially, several kinases may phosphorylate MLKL, but only receptor-interacting protein kinase 3 (RIPK3) has been described while this review was written. MLKL carries several phosphorylation sites, and RIPK3 phosphorylates the so-called “activation loop”. However, complete function of pMLKL requires the depohsphorylation at the hinge region that unleashes the deadly activity of a four-helical bundle (4-HB), which binds PIP2 in the plasma membrane and tends to oligomerize with other pMLKL molecules. Downstream of pMLKL, sensitivity of cells to undergo necroptosis is controlled by proteins that control membrane blebbing and microvesical formation. Unlike previously suggested, pMLKL does not directly form pores in the plasma membrane of cells. Full activation of RIPK3 requires the assembly of the necrosome, a higher order structure that consists of oligomerized RIPK3 molecules that are stabilized by HSP90 and CDC-37, two chaperones the loss of which results in defective necroptosis. Several triggers result in the formation of the necrosome. Death receptor (DR), for example, TNFR1-stimulation in the presence of a caspase inhibitor or a dysfunctional caspase-8 represents the most prominent and best investigated stimulus that requires RIPK1 kinase activity for necrosome formation. RIPK1, as RIPK3, contains a rip homotypic-interacting motif (RHIM)-domain that intercalates with the RHIM of RIPK3 and prevents necrosome assembly. Inhibitors of RIPK1 kinase activity, such as Nec-1s and ponatinib, maintain the inactive state of RIPK3 potentially by keeping RHIM–RHIM interactions intact. Necrosome assembly has been repeatedly reported downstream of Toll-like receptors that bind to the intracellular adapter protein TRIF, which also contains a RHIM domain and activation of this pathway results in robust RIPK1-independent necrosome formation. *In vivo*, reperfusion following ischemic injury severely triggers necroptosis, and several other models have been described, such as injection of recombinant human TNFα into mice. In addition, protein kinase R (PKR) and DAI, a protein that is capable of but functionally not limited to viral DNA sensing activation as triggered by interferons, can activate RIPK1-mediated necrosome formation, but the relative contribution of these two factors remains unclear. Certainly, DAI robustly triggeres necrosome formation via its RHIM domain, possibly via nuclear signalling. However, importantly, necroptosis contributes to acute kidney injury in some models, such as ischemia, but inhibition of necroptosis does not affect other models, such as foliac acid-induced AKI

Necroptosis represents the by far best-studied signaling pathway of RN. Inhibitors of RIPK1 have already reached phase 2 clinical trials^52^, spearheading translational cell death research today^6,53^. It is beyond the scope of this review to mention all aspects known about necroptosis (Fig. 2) and the interested reader may be referred to specialist literature^54–56^. In contrast to apoptosis, a pathway that is dominated by proteases, necroptosis activation depends on the regulation of poly-ubiquitination by E3 ligases, balanced by deubiquitinases (DUBs) and on the activity of kinases, balanced by phosphatases. A tight control of these two systems molecularly decides on the phosphorylation of mixed lineage kinase domain like (MLKL), an essential downstream mediator of necroptosis the phosphorylation of which leads to plasma membrane (PM) attack causing death. This step is indispensable, although not sufficient, to cause cell death and will therefore be used to define necroptosis for the purpose of this review^12^.

Necroptosis can be mediated by DRs, such as tumor necrosis factor receptor 1 (TNFR1), TRAIL and Fas, all belonging to the TNFR superfamily of plasma membrane receptors. The most critical step in the activation of RIPK1, the key kinase in the intracellular signaling cascade downstream of TNFR1, is the loss of K63 and linear polyubiquitin-linkages on RIPK1 that need to be removed to enable the recruitment of pro-caspase-8 to RIPK1, resultant in apoptosis signaling (see above) instead of RIPK1-dependent and RIPK1-independent nuclear factor (NF)-kB activation^57,58^ that leads to a survival signal downstream of TNFR1 when RIPK1 persistently remains polyubiquitinated. The NF-kB signal promotes the expression of cellular FLICE-inhibitory protein, an opponent of caspase-8, resulting in a survival signal rather than apoptosis^24^. Therefore, regulation of linear and K63 linkages is key to the decision of life and death of a cell. Linear ubiquitin chains are assembled by a complex that contains the molecules HOIL1, HOIP and SHARPIN, together referred to as the linear ubiquitin chain assembly complex^59^. Linear chains are removed by the DUB OTULIN and possibly others^60–63^. In contrast, the K63 system is modulated by the DUBs CYLD and A20 to remove linkages controlled by cIAP1 and cIAP2^64–66^.

Upon deubiquitination and together with caspase-8, RIPK1 forms a complex that mediates cellular demise, and the details about the activation of RIPK1 inside this complex, as well as the decision to either signal apoptosis or necroptosis are currently being investigated^67–72^. It appears to be clear that caspase-8 forms homodimers to transduce the apoptotic signal with its unique morphology (see above). However, upon inhibition of caspase-8, either by synthetically generated or viral inhibitors, or in the absence of caspase-8 due to genetic modification *in vivo*, apoptosis can no longer function. It is important to understand that active caspase-8 suppresses the protein RIPK3^9,73^ possibly by cleavage of the protein – to prevent the formation of a RIPK3-oligomer, a huge platform for signaling, referred to as the necrosome that requires the chaperones HSP-90 and CDC-37 to exert its deadly function^74,75^. RIPK3 and three other molecules in the human genome (RIPK1, TRIF and DAI) contain a rip homotypic-interacting motif (RHIM)^76,77^. RHIM domains tend to bind to each other and orchestrate the necroptosis pathway. In an inactive state, for example, the RIPK1–RHIM interacts with the RHIM of RIPK3 to prevent its oligomerization and necrosome formation. The consequence of this function is the prevention of MLKL phosphorylation downstream of the necrosome.^78–80^. Importantly, this model explains the embryonic lethality of RIPK1-deficient mice and RIPK1-deficient delta-RHIM knock-in mice, whereas RIPK1-deficient kinase dead knock-in mice are viable and fertile^81–84^.

However, phosphorylation of MLKL is complex and inconsistent between species. Whereas active phosphorylated RIPK3 inside the necrosome is capable of phosphorylating the activation loop of MLKL, another phosphate between the default protein and the four-helical bundle (4-HB) needs to be removed before the 4-HB can bind to the plasma membrane^85,86^, and it is currently unclear which phosphatase most abundantly exhibits this critical step. Ppm1b has been published to function on pRIPK3^87^, rendering this phosphatase a likely candidate to also regulate pMLKL, but more work is required to understand phosphatases as potential therapeutic targets in the pathway of necroptosis. As soon as the 4-HB of MLKL is no longer properly controlled, it oligomerizes and translocates to cellular membranes, including the plasma membrane. pMLKL is also found inside the nucleus where it might regulate transcription or become functionally altered by nuclear factors^88^. At the plasma membrane, however, pMLKL binds to PIP2^89,90^. The mechanisms that result in plasma membrane rupture downstream of pMLKL-PIP2 binding are entirely unclear today. However, pMLKL is required for necroptosis, but not sufficient as it was recently demonstrated that the ESCRT-III complex regulates necroptosis downstream of pMLKL^91^ (Fig. 2).

Based on the fact that viral caspase inhibitors that target caspase-8 drive the necroptotic machinery, we interpret necroptosis as a pathway that had to be conserved against viruses. In line with this interpretation, the other RHIM domain-containing molecules are also involved to a certain extent in defense against microbes. Toll-like receptors sense bacterial surface markers to signal via the adapter protein TRIF and engage RIPK3 and necrosome formation. Similarly, DAI (also called ZBP1), a protein that contains three RHIM domains, sense viral DNA and possibly other higher order structures^76,77,79,92^. The concept of microbial defense by necroptosis has been extensively reviewed elsewhere^77,93,94^.

### Pyroptosis – gasdermin D-mediated RN

When it was first noticed that macrophages undergo necrotic cell death upon inhibition of caspases, some similarities to necroptosis could have already been noticed, but it took a while to understand that caspase-8 is a key mediator of both of these pathways^95–98^. Inflammasomes sense diverse structures including, but not limited to, microbial surface markers and crystals. On the edge of these higher order structures, caspases can be activated, most prominently caspase-1 and caspase-11. Caspase cleavage of pro-interleukin (IL)-1β and pro-IL-18 results in the intracellular accumulation of IL-1β and IL-18, two long-lasting cytokines that drive systemic inflammation upon release^96,99^. However, despite extensive research on these two cytokines there is no release pathway known to secrete them from the cell unless the plasma membrane ruptures. In parallel to IL-1β and IL-18 processing, these caspases cleave a recently identified key protein of the pyroptosis pathway named gasdermin D^5,11^. The plasma membrane PIP2 is targeted by gasdermin D. This process may last for some hours until a critical concentration of PIP2-bound gasdermin D has accumulated at the plasma membrane, followed by overwhelming membrane extrusions, extensive blebbing and rupture of the plasma membrane^100^. In purified liposomes treatment with purified recombinant gasdermin D, electron microscopy reveals pore-like structures, but this has never been documented for cells in culture. In fact, time lapse videos of pyroptosis^100^ render the pore model of gasdermins unlikely. However, clearly gasdermin D is an indispensable mediator of pyroptosis^98,100–102^. The field of pyroptosis is currently under intensive investigation for a very good reason: it is the most inflammatory cell death known so far. Very recently, a pyroptosis variant has been described in which a family member of the gasdermins, gasdermin E (GSDME) also called DFNA5, results in plasma membrane rupture following cleavage of the GSDME by caspase-3^103,104^. More data are required on GSDME and caspase-3, but if caspase-3 is not exclusively involved in apoptosis, immunohistochemistry for cleaved caspase-3 can no longer be recommended for the detection of apoptosis. In addition, it will be most interesting to investigate the other remaining members of the gasdermin family.

### Interconnection between cell death pathways

In apoptosis, necroptosis and pyroptosis, caspases, proteases, kinases and posttranslational modifications are key to our understanding of this network. Caspase-8 is an example of a central mediator that is involved in all of these pathways. Caspase-3 function may not be limited to apoptosis, but may also be of relevance in GSDME-mediated pyroptosis^103^. Caspases therefore appear to regulate the sensitivity to RN beyond only necroptosis.

Mitochondria appear to be of limited importance in necroptosis^105^, but they are clearly involved in pyroptosis and most likely also in ferroptosis. In the first, they can serve as a platform for the initiation of pyroptotic signaling upstream of inflammasome assembly and GSDMD processing. Apart from that, ferroptosis is different. Lipid peroxidation, iron-catalysed Fenton-type reactions, lipoxygenases and ROS-mediated cell death do not seem to overlap with the remaining cell death pathways. The potential overlap between cell death pathways is of therapeutic relevance as the potentially most effective combination therapy must be chosen upon the consideration of the cell death diversity (see below at 3.3).

## Does the type of cell death define the immunologic response to DAMP release?

**Fig. 3:**
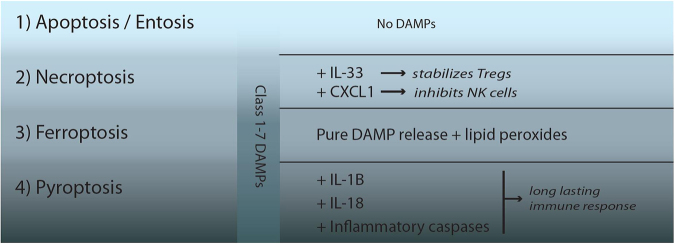
Specific DAMP release defines an inflammatory hierarchy of cell death pathways For a single cell, the mode of cell death does not matter, but it does matter for the environment. The apoptotic program contains several features that prevent its immunogenicity, including the persistence of plasma membrane integrity that prevents the release of DAMPs. We therefore consider apoptosis not at all immunogenic. In contrast to apoptosis, all subroutines of regulated necrosis result in the release of DAMPs because of plasma membrane rupture, and therefore all pathways of regulated necrosis (RN) represent highly immunogenic stimuli. Within the family of RN pathways, modulation of the immune response beyond DAMP release is common and results in a hierarchy of immunogenicity of RN pathways. During necroptosis, IL-33 and CXCL-1 are actively produced in an energy dependent manner, resulting in ST2-mediated stabilization of regulatory T cells and the Mincle-mediated inhibition of innate immunity (e.g., NK cells), respectively. Therefore, necroptosis, apart from releasing DAMPs, inhibits the immune response. During ferroptosis, lipid peroxides may predominate and add an immunogenic component to the DAMPs. However, the most immunogenic RN pathway appears to be pyroptosis due to its active maturation of long-lasting cytokines IL-1β and IL-18 alongside with the release of inflammatory caspases

It is impossible today to induce either apoptosis, ferroptosis, necroptosis and pyroptosis in the very same tissue in a standardized manner in vivo and to directly compare the immune response with these particular events. The potency to stimulate defined populations of immune cells in a specific manner by a given RN subroutine, as estimated from in vitro experiments, and the need for a hypothesis as to why we evolutionary conserved so many different pathways of RN will be the basis for what we call the hierarchy of immunogenicity of RN pathways that will be described in this section (Fig. 3). Importantly, we stress here that all RN pathways, because of their nature of releasing DAMPs, are highly inflammatory and immunogenic per se, and that the differences that will be pointed out here may be minor, but may be of importance in a living organism. Most importantly, the process of dying does not appear to matter for the cell that dies, but obviously matters for the surrounding cells and the homeostasis of the interstitium – in this sense, a cell that is dead is not gone.

### Apoptosis – well defined and well tolerated by the immune system

There are many lines of evidence that suggest that apoptosis is by far the least inflammatory pathway of RCD. First, most physiological cellular turnover during homeostasis in an organism is executed by apoptosis, unless significant genetic changes are artificially introduced. If all of these cells sent out inflammatory signals, life would simply be impossible. Second, apoptotic morphology is very different from necrosis in that the cells shrink, instead of swelling. During the apoptotic program, the cells externalize PS, an eat-me signal for macrophages (see above) that remove apoptotic cells silently and efficiently. Interestingly, caspase activation leads to ROS production in mitochondria that in turn oxidize the potential danger signal high-mobility group box-1 protei thereby preventing its pro-immune activity^106^. With respect to SRN that may be triggered by cells that undergo necrosis, again apoptotic cells are different as caspase activation isolates cells in a functional syncytium from its neighbors by rearranging tight junctions and desmosomal junctions^107^.

### Ferroptosis – pure DAMP release

All pathways of RN release DAMPs and so does ferroptosis. In contrast to necroptosis and pyroptosis (see below), no immune cell-modifying function has been ascribed to ferroptosis, and we therefore interpret ferroptosis today as inflammatory by pure release of DAMPs. However, this simple view does by no means rule out a immunomodulatory role of ferroptosis. It is well possible that peroxidized lipids that are released exclusively from ferroptotic cells trigger the immune system. In addition, ferroptosis might cause oxidation-specific neo-epitopes, such as phosphocholine of oxidized phospholipids and malondialdehyde that have been described and categorized as “class IV DAMPs”^14^. However, in our hierarchy model, we consider ferroptosis as a rather ancient and nonspecific mechanism that does not associate with much immunomodulation apart from DAMP release.

### Necroptosis – modulating immunogenicity

As introduced above, necroptosis is currently interpreted as a second-line defense mechanism against caspase inhibitor carrying viruses that cannot be cleared by apoptosis. Apparently, it is preferable for the organism to remove the virus in an immunologically silent manner if apoptosis is the first choice. However, if caspase inhibition is encountered, no other way to clear the virus appears to exist and DAMP release has to be accepted. However, it is known from cell culture experiments that necroptosis progression, even in very sensitive cells such as L929 fibroblasts, takes at least 3 h following induction until the plasma membrane ruptures. This time window provides enough capacity for an active cell to express proteins. It has been described that the chemokines CXCL-1 is produced during necroptosis and that this molecule inhibits NK cells through its receptor Mincle^108,109^. Although this mechanism has been described as a means for pancreatic cancer cells to prevent the immune infiltration, it is conceivable that CXCL-1 also limits the immune response following DAMP release by necroptosis. Along these lines, the cytokine IL-33 is actively produced during necroptosis and has been termed the “necroptotic DAMP”^81^. Although possibly not specific for necroptotic cell death, the alarmin IL-33 has been shown to stabilize regulatory T cells via ST2-receptors, allowing them to adapt to an inflammatory microenvironment^110,111^. In addition, pMLKL functions as an upstream activator of the inflammasomes resulting in the maturation of IL-1β, which may be released during necroptosis execution^112,113^, even in the absence of gasdermin D (see 'Pyroptosis – more inflammatory than pure DAMP release'). Therefore, bearing in mind that all necrotic signaling pathways do release pro-inflammatory DAMPs and therefore are capable of cross priming, we speculate that necroptosis results in less systemic inflammation than uncontrolled DAMP release and other RN pathways.

### Pyroptosis – more inflammatory than pure DAMP release

In pyroptosis, both the maturation of long-lasting pro-inflammatory cytokines IL-1β and IL-18 and the process of gasdermin D-mediated plasma membrane rupture are coupled to the same proteases, presumably caspase-1 on the edge of inflammasomes. Apparently, upon inflammasome engagement, the organism benefits from reacting with a systemic immune response. We therefore consider pyroptosis the most inflammatory cell death pathway.

## Harnessing RN for therapy

The nature of AKI on intensive care units has little in common with cell culture experiments or mouse models of IRI, cisplatin- or folic acid toxicity, rhabdomyolysis and other models that are popular to study AKI in vitro or in vivo. There is no preclinical model of what clinicians experience on the ICU: a continuously hypoperfused organ with a significant variation of blood pressure and glomerular filtration within hours following catecholamine treatment. In this highly complex ICU setting, patient kidneys are likely to experience ongoing disease stimulation. Translated to cell death research, synchronized necrosis of functional units may happen every other minute over days until renal function declines to a level in which dialysis becomes inevitably necessary. This picture is accompanied by acute tubular necrosis in the decreasing amounts of urine that these kidneys are capable of producing.

### Inhibition of necroptosis

Inhibiting necroptosis using pharmacological tools was logically expected to efficiently block relevant disease processes. However, only a very few in vivo models are reproducibly protected by RIPK1-inhibition (e.g., by necrostatin-1, Nec-1), or by genetic modification of RIPK3. Very few data exist for MLKL-ko mice, but it is important to use this tool as RIK3-deficient mice are not exclusively deficient for necroptosis, but also lack RIPK3-dependent signalling that is necroptosis independent^8,114,115^ and still might contribute to the protection from some in vivo models. In addition, the first published inhibitor of necroptosis (Nec-1)^116^ represents unique pharmacological properties as it functions as a ferrostatin^117^ with intrinsic anti-necroptotic activity on a hydantoin backbone^16,118^. Therefore, some of the beneficial effects may be due to prevention of ferroptosis rather than necroptosis. Corrected for all these concerns, necroptosis appears to be important in IRI because RIPK3-deficient mice are protected from IRI models in the heart^82^ and the kidney^119^, and RIPK3-deficient mice exhibit superior survival rates following injection of recombinant TNFα^82,120,121^. More specific kinase inhibitors of RIPK1 (Nec-1s and the RIPK1/RIPK3 inhibitor ponatinib) are beneficial in the latter model^122,123^.

### Inhibition of ferroptosis

With respect to prevention of AKI, in our hands, no single compound has been more effective than the small-molecule ferrostatin 16–86^17,42,124^. For all models of IRI tested so far *in vivo*, no comparably effective protection has been achieved in isolated renal tubules or AKI models so far. In all, 16–86 has been developed chemically in a series of modifications from the original compound Fer-1 that was found in a screen of small molecules to protect HT1080 cells from erastin-induced ferroptosis^2^. Because of its general availability, most ferroptosis projects today use this first-generation ferrostatin for investigations in vitro. However, it should be pointed out that the efficacy is almost completely limited to in vitro studies, and the in vivo stability in plasma and liver liposomes is short, and therefore unlikely to yield the strong effect that is expected when ferroptosis is prevented effectively. Despite this knowledge, effects of Fer-1 in vivo have been detected, but they are likely underestimating the beneficial potential, and those experiments should be re-evaluated as soon as more stable and more potent ferrostatins become available. One such compound that has been intermediately used to prevent ferroptosis is referred to as 11–92, a single charge preparation from the Stockwell laboratory. In all, 11–92 was tested *in vivo* in models of Huntington's disease and periventricular leukomalacia (PVL) and in the ex vivo model of isolated renal tubules that underwent hypoxia/reoxygenation and iron/hydroxyquinolone. This was also the first study to propose a direct inhibitory effect on lipid reactive oxygen species generation by ferrostatins^43^. Another small molecule, liproxstatin-1, was found in parallel to prevent IRI in the liver model, and was effective in reversing the detrimental renal tubular phenotype of inducible GPX4 depletion^117^. However, liproxstatin-1, 11–92 and 16–86 are preliminary in terms of plasma stability and further compounds with better pharmacokinetics are currently under development. Among the most recent developments, Krainz et al.^125^ followed a novel approach employing mitochondrial-targeted nitroxide as a potent inhibitor of ferroptosis, introducing several compounds (e.g., XJB-5-131 and JP4-039) that function as nitroxide-based lipid peroxidation mitigators, the biological activity outperformed all other ferrostatins, confirming the central role of mitochondria within the ferroptosis pathway (Fig. 1). Nonetheless, neither XJB-5-131 nor JP4-039 has been tested in animals. Ironically, the best studied ferrostatin is Nec-1 – see below.

### Treatment of RN requires combination therapy

As with many successful therapies that are currently used in nephrology, such as the treatment of hypertension, immunosuppression, antibiotic therapies and many others, prevention of RN will be unlikely achieved by monotherapy unless ferrostatins are developed that contain intrinsic anti-necroptotic activity, such as one of the very first cell death blockers, Nec-1 incidentally does^117^. However, Nec-1 is a hydantoin, and hydantoins may result in severe side effects during phase IV clinical trials, restricting pharmaceutical companies from promoting hydantoins.

Targeting two cell death pathways simultaneously in the context of ischemic injury revealed an overwhelming protective effect^119^, surpassing the sum of each single compound or genetic knockout. This suggested that RN pathways may communicate, as pyroptosis and necroptosis obviously do through the common key player caspase-8, which on top of this is also involved in apoptosis. In the very early days, targeting apoptosis alone is another example of communicating cell death pathways, and only by the addition of zVAD-fmk, a broad spectrum caspase inhibitor, necroptosis has been identified in vitro^126^and in vivo^127^. However, administration of zVAD-fmk to in vivo models did not result in any measurable protection^128^, resulting in the conclusion that apoptosis might not contribute much to the pathophysiological course of AKI. However, zVAD-fmk is not a very good inhibitor of intrinsic apoptosis downstream of mitochondrial outer membrane permeabilization and cytochrome C release. Novel caspase inhibitors, such as emricasan (IDN-6556)^129,130^ might answer this long standing question.

In conclusion, we consider it unrealistic to ever prevent the first hit that the kidneys have to overcome upon resuscitation, cardiac surgery, systemic infection etc. If a clinical trial will be designed employing cell death targeting drugs, for example, ferrostatins, siRNA against p53, Necs, complement inhibition, next-generation caspase inhibitors or combination therapies, it needs to be a progression trial.

## Prevention of autoimmunity by removal of necrotic debris

Once a cell dies by necrosis inside an organism, it is not lost. It will be recycled to save energy. The mechanisms of necrotic debris uptake are ancient and can be evolutionarily followed back to single-cell organisms. Bits and pieces of the necrotic mass are sensed by unknown means and endocytosed into single membrane vesicles, in a process that depends on the intracellular molecule rubicon^131^. Sorting of these vesicles inside the intracellular compartment requires the fusion with lysosomes to breakdown and recycle their valuable cargo in a process that is comparable to the fusion of autophagosomes with lysosomes during the process of autophagy^132^. The microtubule-associated protein-1 light chain 3 (LC3) translocates and becomes conjugated to the cellular lipid membrane as a key step^133^, and this complex membrane modification is mediated by a machinery that requires proteins such as ATG5, ATG7, Beclin-1 and ATG16L. These proteins are essential for autophagy and LC3-associated phagocytosis (LAP – synonymously referred to as “non-canonical autophagy”), the process that recycles necrotic cell debris. The processes of autophagy and LAP both provide energy sources to a cell, it is therefore likely that they also share evolutionary conserved pathways. However, autophagy does not require rubicon, but depends on a pre-initiation complex that consists of proteins such as FIP200, ULK1 and others.

Genome-wide association studies have mapped susceptibility loci for Crohn´s disease, ulcerative colitis and systemic lupus erythematosus (SLE) to several of the molecules involved in both LAP and autophagy^134^, and because LAP has been only recently discovered it was widely believed that defects in autophagy represent the mechanistic site important for the development of these autoimmune diseases. In addition, it was known that autophagy represents a “renoprotective” mechanisms^135,136^, at least during the very early stages following AKI^137^, but this is currently a matter of debate^135,136,138^. It is clear now that the loss or pharmacological inhibition of other proteins that are required for the fusion of autophagosomes/LAP vesicles with the lysosomes, such as acid sphingomyelinase, results in increased disease sensitivity to IRI and a stronger immune response to the target tissue^139^.

**Fig. 4:**
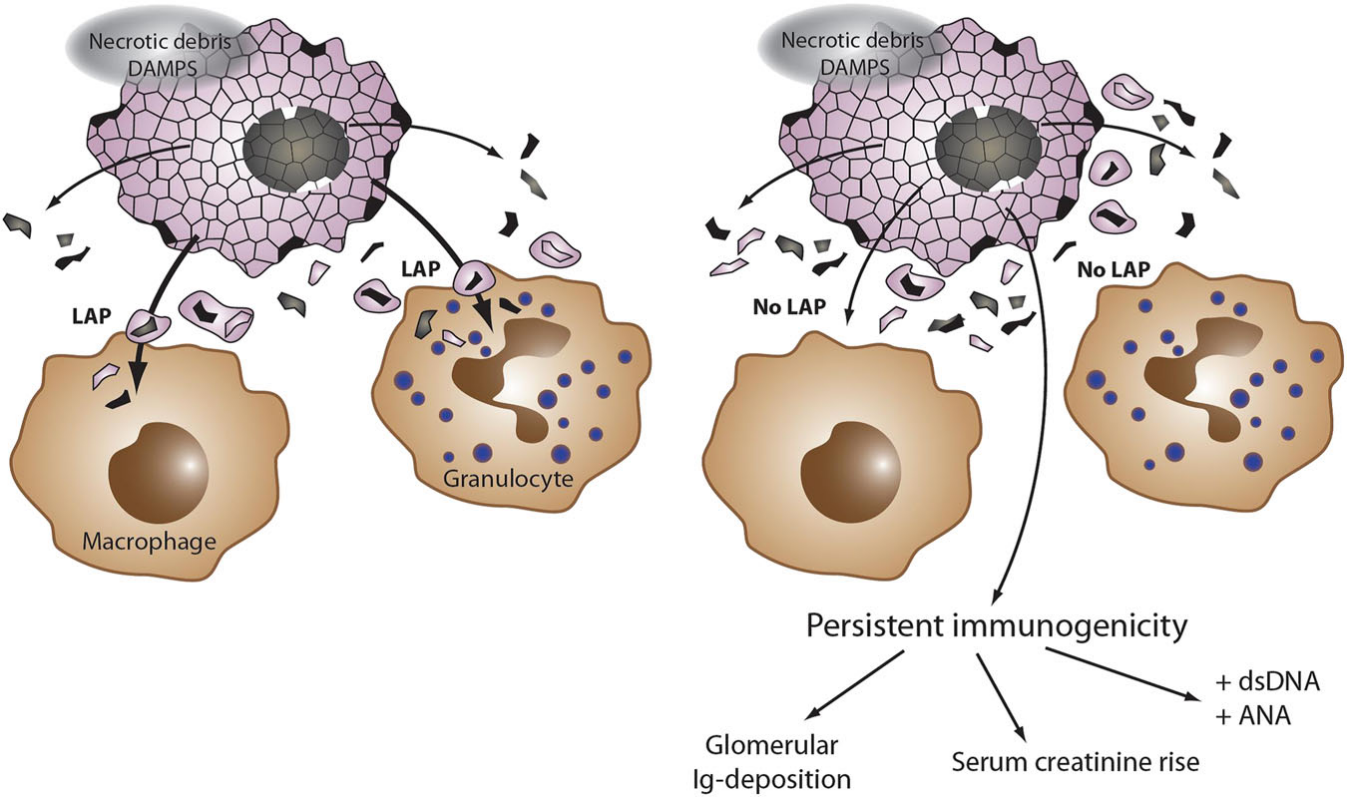
Failure to remove necrotic debris results in autoimmunity Macrophages and granulocytes remove necrotic debris by a process of LC3-associated phagocytosis (LAP), also referred to as noncanonical autophagy. Failure to remove necrotic debris results in persistence of DAMP-mediated immunogenicity. Over the time of months to 1 year, mice deficient in LAP (rubicon-ko mice) develop a lupus-like disease including immunoglobulin deposition in glomerula, increased serum levels of creatinine and urea, and development of autoantibodies against double-stranded DNA and antinuclear antibodies (ANAs)

Necrotic debris contains highly inflammatory components, such as damaged mitochondria, lysosomes that lost their membrane integrity and release highly active proteases, peroxisomes that carry enormous amounts of higher order reactive oxygen species, nuclei that release histones, which trigger neutrophils and hundreds of other components, including autoantigens such as double-stranded DNA. Obviously, removing these highly inflammatory structures in an efficient and immunologically silent manner is important. Data now suggest that lack of LAP in LysM-cre-expressing cells, such as monocytes and macrophages, results in a slowly progressing SLE-like disease in mice. Features of AKI have been demonstrated including immunoglobulin deposition in the glomerula, elevation of serum creatinine and serum urea levels, and antinuclear antibodies, as well as antibodies against double-stranded DNA have been detected^140^. In the same study, conditional deletion of FIP200 or ULK1 did not result in SLE-like phenotype, whereas deletion of ATG7 and Beclin-1 did. In conclusion, defects in LAP and therefore failure to remove necrotic debris results in autoimmunity (Fig. 4) whereas autophagy, at least in LysM-cre-expressing cells, appears to be dispensable for this process.

This novel concept may have important implications for both AKI and kidney transplantation, because necrosis drives the immune response in both of these settings. Failure to remove necrotic debris might result in a persistent immune response also following AKI, and result in the generation of donor-specific antibodies and rejection (see section 'Specific considerations on renal cell death' below) following transplantation. This effect may be highly augmented if necrotic cell death subroutines such as pyroptosis are involved. However, the above-mentioned investigation deleted rubicon and other proteins of this pathway from macrophages^140^, the predominant cells to undergo pyroptosis, and therefore the results may be underestimating the severity of the in vivo effect that we might experience in transplant settings or ischemic and toxic AKI.

## Specific considerations on renal cell death

Given (i) the complexity of the renal microenvironment and (ii) the consequences of RN on the surrounding microenvironment, cell death in the kidney is best interpreted by the organ as a whole, and not by tubular, glomerular, interstitial or endothelial compartments in an isolated manner. Even beyond the organ itself, systemic effects related to DAMP release have been described in distant organs in response to AKI, and nicely highlighted in a recent mouse study examining the lungs following renal transplant surgery. These authors found diverse pathways of RN affecting lung endothelial cells to be driven into cell death by DAMPs released from the necrotic cells of the kidney, identifying necroptosis, ferroptosis and others in this tissue^141,142^.

In the human situation, the detection of cell death will be even more difficult because of pre-existing comorbidities that are hardly ever considered in classical nephrological in vivo research.

Apart from AKI, which will be discussed in more detail in the following section, it should be mentioned that cell death in the kidney may be of importance for several other conditions, such as necrotizing glomerulonephritis, for example, during anti-neutrophil cytoplasmic antibody (ANCA) vasculitis and other forms of rapid progressive glomerulonephritis.

### RN in AKI

The effects of genetically or pharmacologically inhibiting more than one pathway of RN in the same *in vivo* model at the same time was first described for models of AKI (cisplatin toxicity and IRI) and provided striking levels of organ protection^42,119^. As a result of the interconnectivity of cell death subroutines (compare with section 'Interconnection between cell death pathways'), it appears that inhibition of more than one cell death subroutine leads to more protection than expected, suggesting that combination therapy may potentiate the beneficial effects. However, it is of paramount importance that basic science nephrologists and cell death researchers focus to identify the relative contribution of each pathway of RN to identify the best pharmacological target, and succeed in first clinical trials^143^.

MPT-RN, which is partially mediated by the mitochondrial molecule cyclophilin D and has recently been suggested to be mediated by the c-subunit ring of the F1FO ATP synthase^144^, represents yet another cell death subroutine that is likely overlapping to a minor extent with ferroptosis. MPT-RN has been suggested to contribute to AKI^145^ and other models of ischemia-reperfusion, for example, in the heart^146,147^. Prevention of MPT-RN in IRI in mice resulted in significant protection from functional markers of AKI, but it did not reach the protective level provided by inhibition of necroptosis^119^. In contrast, either pharmacological interference with necroptosis or RIPK3 deficiency was less protective compared with inhibition of ferroptosis with a third-generation ferrostatin referred to as 16–86^42^. Based on these observations, we today interpret ferroptosis as the most prominent contributor to AKI, also because of its unique morphology in acute tubular necrosis, termed SRN. SRN is best investigated in microperfused renal tubules^42^ where ferroptosis is induced by erastin added to the perfusate. SRN appears to always originate from a single cell that dies in the functional unit of a renal tubule and that from this site of origin, the necrosis spreads from cell to cell, always affecting the neighbor, but never a cell that is distant in the tubule. Intracellular NADPH abundance may explain the progress of this chain reaction^39^ where endothelial cells kill their neighbors that are connected to them via tight junctions. In that model, loss of buffering capacity (NADPH, NADH etc.) gets lost in all cell death pathways investigated so far, might results in an exchange between cells in the functional epithelial layer, exchanging their buffer capacity according to concentration gradients. If the gradient gets steep toward a dying cell, neighboring cells will provide NADPH in an attempt to compensate for the dying cell next to them^39,40^. However, upon plasma membrane rupture of the dying cell, the neighbors contain the lowest possible buffering capacity, setting these cells at highest risk to ferroptosis. This model needs to be investigated in more detail, but currently provides a working hypothesis on how a necrotic cell might drive others into ferroptosis, an observation that might also be of importance considering the pathophysiology of stroke or myocardial infarction. Efforts are now being undertaken to monitor necrosis progression in hearts and brain by means of intravital microscopy.

### Cell death in transplant rejection

Almost two-thirds of all renal allografts are thought to be lost due to antibody-mediated rejection (ABMR), in most cases a slowly progressing transplant disorder that accounts for chronic transplant rejection^148^. In contrast to acute or subacute T-cell-mediated rejection that is easily treated with high-dose steroids for a couple of days in most cases, no effective treatment exists to target ABMR^149^. The nature of this disease is largely elusive, and the pathophysiological course remains to be clearly demonstrated. Attempts to treat ABMR in transplanted patients are frustrating as plasma exchange in most cases does not provide significant benefits for much longer as the plasma exchange is effectively performed. Anti-CD20 antibodies are often applied, but other than pre-plasmablasts, highly differentiated plasma cells, the indirect mediators of the disease that are hidden in bone marrow niches, do not express CD20 and are not targeted by rituximab^150^, yet the risk for chronic and acute infection increases beyond the already high levels of transplanted patients. However, memory B cells and donor-specific antibodies (DSA) appear to be of central importance in this disease and memory B-cell priming may occur in the presence of immunosuppression. Given the strong increase of cases of ABMR upon tapering of the standard immunosuppression^151^, an intriguing hypothesis emerges that might add to our understanding of ABMR and provide a preventive rationale.

**Fig. 5:**
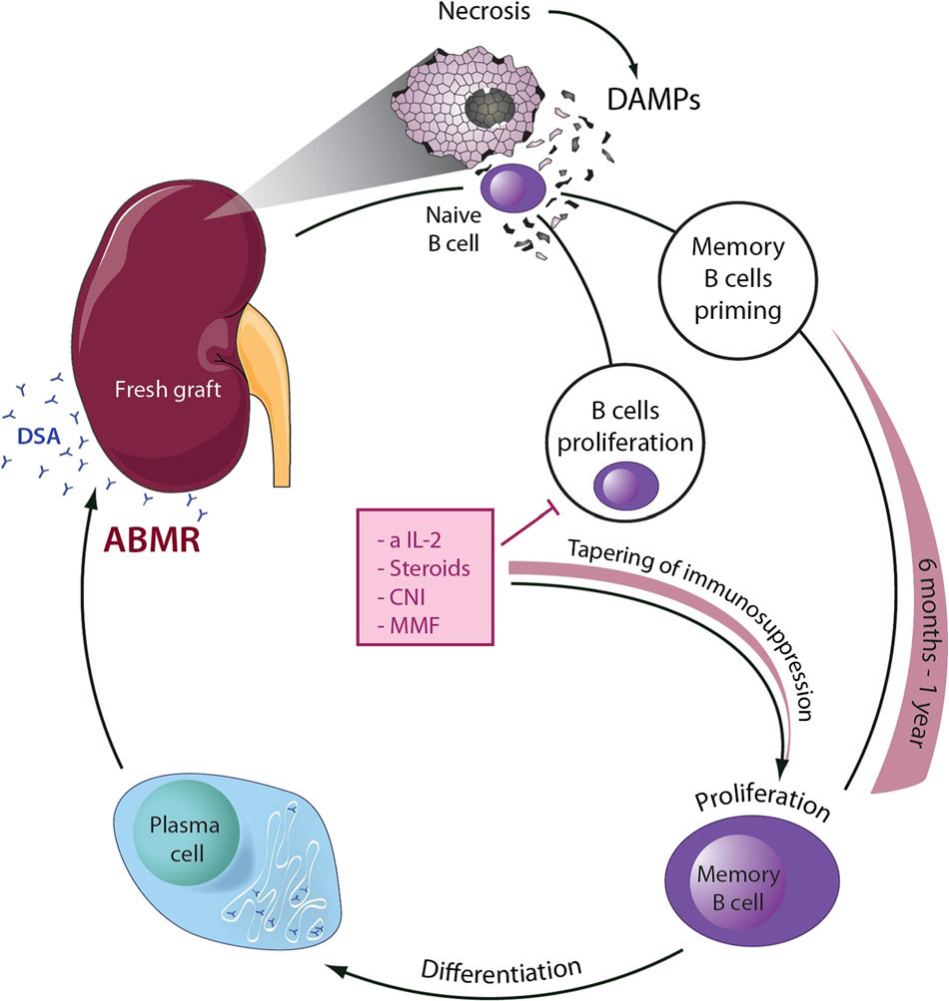
A novel hypothesis for the development of antibody-mediated rejection (ABMR) Upon the process of transplantation of damaged (e.g., marginal) organs, naive recipient B cells encounter massive necrosis and DAMPs during the very first passage through the graft and are being potently primed. The proliferative B-cell response and the subsequent differentiation to plasma cells is prevented by the standard immunosuppression at this early time point. However, upon tapering of the immunosuppression during the first year after virtually successful transplantation, primed memory B cells progressively proliferate and terminally differentiate into plasma cells, producing donor-specific antibodies and mediating ABMR. Clinically, at this stage, plasma exchange may temporarily prevent ABMR progression, but as soon as plasma exchange stops, ABMR will slowly reactivate. Our model suggests that early interference with regulated necrosis unlike HLA- or even blood group-matching possesses the capacity to prevent memory B-cell priming and ABMR. Addition of necrosis blocking agents, for example, small molecules in the machine perfusate, are predicted to prevent ABMR

Many kidneys transplanted today are either taken from brain dead donors, non-heart beating donors or are classified as marginal kidneys for other reasons. All of these conditions relate to significantly increased necrosis in the tubular system, and maybe more importantly also to RN in resident macrophages and dendritic cells. Whereas necrotic cell death in renal tubules is clearly dominated by ferroptosis^42^, resident immune cells of the donor may undergo other forms of RN^53^, including highly inflammatory pyroptosis in macrophages. In a scenario like this, in which both resident immune cells and parenchymal cells release DAMPs and pro-inflammatory cytokines, naive recipient B cells that follow the blood stream into the transplant may become exposed to the strongest possible trigger to prime components of the adaptive immune system. A trigger like this may well exceed stimulation by HLA- and even blood group incompatibility. However, proliferation and clonal expansion of B cells is prevented in this scenario^152^ given the standard quadruple immunosuppression that includes targeting of CD-52, CD25 and CD3 as “induction therapy”, accompanied by steroids, calcineurin inhibitors and mycophenolate mofetil. Importantly, these drugs do not appear to affect priming of naive B cells (Fig. 5).

Upon tapering of the immunosuppression, long lived memory B cells may initiate clonal expansion and terminal differentiation into plasma cells that now result in high levels of DSA and ABMR. It is a fascinating observation that even in blood group incompatible living donors that are not at all human leucocyte antigen (HLA) matched, ABMR appears to be less common than in deceased donors that have been perfectly matched according to the Eurotransplant algorism^153^, and it has recently been confirmed that DSA require a pre-activated immune system to affect renal transplants^154^. Importantly, the recognition of donor DAMPs is not limited to adaptive immunity, but is also sensed by monocytes that trigger acute rejection^155^. We conclude that both ABMR and acute rejection are triggered by necrotic debris and that prevention of DAMP release by addition of ferrostatins, necrostatins and other RN-targeting compounds may be effective to prevent both acute and chronic rejection, including ABMR in deceased donor transplantation.

## Conclusions

In conclusion, RN contributes to several renal diseases. These include AKI, ABMR and autoimmune disorders. With our growing knowledge of the relative contribution of diverse signalling pathways of RN, it is now possible to define novel therapeutic targets. To address these unmet clinical needs, inhibitors of RN (necrostatins, ferrostatins etc.) are currently optimized by medicinal chemists for safety, liver liposomal stability, plasma half-life and potency. Some necrostatins have already entered clinical trials for the treatment of autoimmune diseases. Hopefully, with the current knowledge and the help of pharmacological companies or investigative consortia that conduct clinical and associated mechanistic studies to improve outcomes for transplant recipients and patients who suffer from AKI, the translation to clinical routine will be made possible.
